# The relationship of job and life satisfaction with symptoms of anxiety, depression, and irritability/anger in nurses

**DOI:** 10.3389/fpubh.2025.1617148

**Published:** 2025-10-13

**Authors:** Bogusława Serzysko, Renata Mroczkowska, Jolanta Kamińska, Beata Podsiadło, Grzegorz Żarczyński, Anna Krajza

**Affiliations:** ^1^Department of Healthcare, Higher School of Applied Sciences in Ruda Śląska, Ruda Śląska, Poland; ^2^Institute of Health Sciences, University of Opole, Opole, Poland; ^3^Faculty of Medicine and Health Sciences, Academy of Applied Sciences in Nowy Sącz, Nowy Sącz, Poland; ^4^Silesian Medical University in Katowice Faculty of Health Sciences in Katowice, Katowice, Poland; ^5^College of Health Sciences in Bydgoszcz, Bydgoszcz, Poland; ^6^Maria Sklodowska-Curie National Institute of Oncology, National Research Institute Gliwice, Gliwice, Poland

**Keywords:** nurses, job satisfaction, life satisfaction, anxiety symptoms, depression symptoms, irritability/anger symptoms, mental health, occupational stress

## Abstract

**Introduction:**

The nursing profession involves substantial emotional and organizational demands, which may negatively affect nurses’ mental health and overall satisfaction with life and work.

**Objective:**

To assess the levels of anxiety, depression, and irritability/anger symptoms in nurses and examine their associations with life and job satisfaction. In this study, the HADS-M subscale assessing irritability/anger was used. It captures symptoms of irritability and anger but does not measure aggression or anger expression as behavioral constructs.

**Methods:**

A cross-sectional online survey was conducted among 538 registered nurses in Poland. Validated instruments were applied: HADS-M (for anxiety, depression, and irritability/anger symptoms), SWLS (life satisfaction), and SSP (job satisfaction). Statistical analyses included descriptive statistics, Spearman’s rank-order correlations, internal consistency (Cronbach’s alpha), and multiple linear regression (two-tailed, *α* = 0.05).

**Results:**

A high prevalence of psychological symptoms was observed. All symptom domains were significantly and negatively correlated with life and job satisfaction (*p* < 0.001). Multiple regression analyses indicated that depressive symptoms were the strongest predictors of both life satisfaction (*β* = −0.429) and job satisfaction (β = −0.315), followed by irritability/anger symptoms. All models were statistically significant (*p* < 0.001).

**Conclusion:**

Anxiety, depression, and irritability/irritability/anger symptoms significantly reduce nurses’ satisfaction with life and work. Depressive symptoms exerted the most profound impact. These findings emphasize the urgent need for targeted mental health support programs and preventive strategies to enhance nurses’ well-being and professional performance.

## 1 Introduction

Nurses play a pivotal role not only in providing direct patient care but also in shaping standards of care and advancing the professional development of nursing as a discipline (1, 2). They constitute the largest professional group in the Polish healthcare system. According to data from the Supreme Chamber of Nurses and Midwives, by 2022, 36% of actively employed nurses were aged 51–60, and nearly one-third were eligible for retirement. The average age of a nurse in Poland was 54 years and is projected to increase to 58 years by 2030 (3).

The majority of nurses work in rotating shifts, which disrupt circadian rhythms and adversely affect both mental and physical functioning (4). Nursing is considered a profession with a high risk of exposure to occupational stressors. In addition to shift work, the most frequently cited burdens include exposure to death and dying, emotional and physical overload, staff shortages, workplace bullying, and professional burnout (5, 6). The cumulative effect of these factors may result in decreased quality of life and reduced job satisfaction.

Subjective quality of life is a key component of psychological well-being (7, 8). Research indicates that chronic stress, emotional exhaustion, and negative affective states-frequently experienced by nurse-may diminish the quality of care, professional performance, and job satisfaction (9). Emotional responses such as anxiety, worry, and irritability are natural elements of daily functioning; however, prolonged stress exposure can result in psychophysiological dysfunctions, including sleep disturbances, somatic complaints, cardiovascular issues, or substance abuse (10–20). Shift work further impairs the gastrointestinal, hormonal, and cardiovascular systems (21–23). Accordingly, the World Health Organization recognizes nursing as one of the most demanding and stressful professions (24).

Studies conducted by researchers such as Zheng et al. (25), Desouky and Allama (26), Niedhammer et al. (27), and Nakada et al. (28) confirm high prevalence of depressive symptoms among nurses. Zheng et al. (25) additionally demonstrated that these symptoms are often intensified in this professional group. Studies conducted among nurses in Iran also confirm the frequent occurrence of anxiety and depressive disorders (29, 30). Abadi et al. (31) examined the influence of personality and occupational factors on the prevalence of anxiety and depression. Both physical and mental health in nurses are closely linked to the quality of their professional performance—particularly in relation to patient care, job satisfaction, and efficacy.

In recent years, growing attention has been paid to anger symptoms and irritability as important indicators of psychological strain among nurses. Although not always manifesting as overt aggression, these symptoms are often referred to in the literature as irritability (internalized anger) and may reflect difficulties in emotional regulation. Studies indicate that trait anger and modes of anger expression—particularly emotional suppression—are associated with lower job satisfaction and greater risk of burnout. Additionally, nurses frequently experience aggression in the workplace, both from patients and colleagues, which significantly compromises their psychological well-being. Such experiences may lead to chronic overload, reduced engagement, and lower job satisfaction (32–38).

Today’s nursing work environment also involves a risk of direct emotional aggression from patients or their families. Such encounters may result in chronic stress, anxiety, emotional tension, and overall deterioration in well-being. In severe cases, they may lead to post-traumatic stress disorder (PTSD) (38). In the face of such threats, job motivation, satisfaction, and care quality may deteriorate, while the risk of medical errors and adverse events increases (20).

To better understand these associations, this study adopted the Job Demands-Resources (JD-R) model, which posits that occupational well-being depends on the balance between job demands (e.g., workload, emotional pressure) and resources (e.g., support, autonomy, recognition) (39, 40). When demands outweigh resources, the risk of burnout and emotional dysregulation increases. This approach is complemented by the Job Demand—Control model (41), which emphasizes that the greatest risk of occupational stress occurs when high workload is accompanied by low decision latitude (42). Siegrist’s effort–reward imbalance model (43) further suggests that insufficient rewards—both financial and emotional—in the context of high employee effort contribute to chronic stress and depressive symptoms (44). On the individual level, the theory of emotional self-regulation (Baumeister and Heatherton) (45) proposes that chronic stress weakens the ability to control emotions, which may lead to irritability and a decline in quality of life (46).

Recently, increased scholarly attention has been devoted to anger symptoms-often labeled “irritability” or “nervousness” in the literature. Although these symptoms are not unequivocally classified as clinical aggression, they constitute important indicators of emotional overload and affect regulation difficulties. Studies have shown that trait anger and its expression patterns, such as emotional suppression, are significantly associated with reduced job satisfaction and an increased risk of burnout among nurses (47–49). Furthermore, exposure to workplace violence—both verbal and physical—from patients, their families, or coworkers—can impair psychological well-being and lead to burnout symptoms, reduced engagement, and lower job satisfaction (33–35).

Despite numerous studies confirming the high level of emotional strain among nurses, there is still a lack of comprehensive analyses that simultaneously assess the relationship between the severity of negative emotions—such as anxiety, depression, and anger symptoms—and quality of life, both professionally and personally, in this occupational group. This is particularly true for studies that incorporate a broad range of psychosocial and organizational variables in the context of the Polish healthcare system.

International studies by Liu et al. (33) and Kim et al. (47) confirm the associations between symptoms of anxiety, depression, and anger symptoms and professional burnout and quality of life among nurses, but most often focus on a single variable or individual risk factor. Similarly, researchers such as Zheng et al. (48), Niedhammer et al. (49), and Nakada et al. (50) report a high prevalence of depressive and anxiety symptoms in this occupational group, highlighting the global nature of the problem. However, in Poland, there is still a lack of studies addressing the co-occurrence of multiple negative emotions and their cumulative impact on nurses’ well-being—both at work and beyond. Previous analyses, such as those by Uchmanowicz et al. (51) and Talarowska and Gałecki (52), have rarely explored the relationship between the full spectrum of negative emotional states and quality of life in the specific context of a healthcare system characterized by chronic staffing shortages, work overload, low salaries, and organizational changes.

To avoid conceptual ambiguity, it is necessary to clarify the main constructs examined in this study. Job satisfaction can be understood as the individual’s evaluation of their professional duties, reflecting the extent to which these activities are perceived as meaningful, rewarding, and consistent with personal values (53). Life satisfaction, in turn, represents a broader perspective, referring to the overall cognitive judgment of the quality of one’s life as a whole (54).

In contrast to these cognitive appraisals, the study focuses on emotional symptoms. Symptoms of anxiety are conceptualized as persistent tension, worry, or apprehension that interfere with daily functioning. Symptoms of depression do not necessarily indicate a clinical disorder but rather reflect mood-related difficulties, including sadness, loss of interest, or reduced motivation. Anger is conceptualized as a natural, immediate emotional reaction to frustration or provocation; in the present study we operationalize this affect as “anger symptoms” using the HADS-M *irritability/anger* subscale. Unlike aggression, which refers to behavioral manifestations, anger symptoms may serve an adaptive function if adequately regulated (10, 45).

It is also important to distinguish between mood-related phenomena (such as symptoms of anxiety and depression), which are generally more enduring and pervasive, and emotions like anger, which are typically shorter in duration and arise in response to specific stimuli (10). This distinction underscores the complex and multidimensional nature of psychological functioning considered in the present research. For clarity, in this manuscript we treat anxiety and depressive symptoms as mood phenomena (longer-lasting, pervasive), whereas anger is treated as an emotion (short-lived, stimulus-bound); we assess anger symptoms using the HADS-M anger subscale and do not assess aggressive behavior. Throughout this manuscript, the term “depression” refers exclusively to “symptoms of depression” as measured by the HADS-M; we do not infer clinical diagnoses.

The aim of this study was to evaluate the associations between the intensity of anxiety, depressive, and irritability/anger symptoms (operationalized via the HADS-M irritability/anger subscale) and levels of life and job satisfaction among nurses. The study also considered occupational factors such as years of experience, duration of employment at the current facility, shift work schedule, average monthly working hours, and number of jobs held. While stress and occupational burnout are addressed in the theoretical framework of this study, they were not directly measured and are discussed in the limitations section.

## 2 Materials and methods

### 2.1 Study design and setting

This was a cross-sectional study conducted using a diagnostic survey method. Data were collected via an anonymous online questionnaire distributed through the Google Forms platform between June 2023 and February 2024.

Given the specific characteristics of nursing work and the difficulty of conducting on-site research in clinical settings, a remote data-collection approach was adopted. Recruitment relied on purposive sampling via closed professional groups and thematic forums on social media, enabling access to nurses employed across multiple regions of Poland and in diverse healthcare facilities (hospitals, outpatient clinics, long-term care institutions, and other medical settings). Geographic quotas were not applied and province/voivodeship was not recorded; therefore, the study does not claim nationwide representativeness. This stance is consistent with the size and structure of the national nursing workforce reported by the Supreme Chamber of Nurses and Midwives (NIPiP) in 2022 (3), against which our convenience sample should not be viewed as representative.

Participation was entirely voluntary; no directly identifiable information was collected; and all data were stored securely with access limited to the research team. This strategy prioritized feasibility and confidentiality for a hard-to-reach workforce while acknowledging the non-probability nature of the sample.

Although not designed for population inference, the sample provided adequate statistical power for within-sample associations. A post-hoc power analysis in G*Power 3.1 for a two-tailed correlation test (Spearman), with *α* = 0.05, assumed medium effect (*ρ* = 0.30), and *N* = 538 yielded power 1–*β* ≈ 0.999, indicating very high sensitivity to detect the hypothesized relationships.

### 2.2 Participants

The study targeted actively employed registered nurses in Poland. Inclusion criteria were: current employment as a nurse in Poland, age ≥ 18 years, ability to complete an online Polish-language survey, and informed, voluntary consent with acknowledgment of the study’s purpose, anonymity, and data-processing procedures.

Exclusion criteria were: not currently employed as a nurse, nursing students/interns, midwives or other healthcare professionals working in non-nursing roles, lack of consent, and duplicate or incomplete questionnaires (removed prior to analysis).

Participants were recruited via purposive sampling through closed professional groups and thematic forums on social media. Respondents completed the questionnaire independently in a web browser. No geographic quotas were used and province/voivodeship was not recorded; therefore, we do not claim nationwide representativeness.

A total of 550 forms were submitted. After eligibility screening and data-quality checks, 538 fully completed questionnaires were retained for the final analysis. The sample encompassed nurses with diverse demographic and professional profiles, including variation in age, years of experience, employment setting (e.g., outpatient care, medical/surgical wards, ICU/ED, long-term care), work schedules, and educational levels.

### 2.3 Population characteristics

The final sample comprised 538 nurses. The majority were women (96.65%, *n* = 520), while men accounted for 3.35% (*n* = 18). The largest age group was 51–60 years (40.15%, *n* = 216), followed by 41–50 years (31.78%, *n* = 171). The youngest participants, aged 22–30, represented 9.29% (*n* = 50), and those over 60 years accounted for 6.32% (*n* = 34).

Most participants lived in urban areas (76.77%, *n* = 413), while 23.23% (*n* = 125) resided in rural areas. In terms of education, the most frequent qualification was a bachelor’s degree in nursing (39.41%, *n* = 212), followed by a master’s degree (33.83%, *n* = 182) and secondary medical education (26.77%, *n* = 144). Regarding marital status, most respondents were married (67.47%, *n* = 363). Others were single (10.22%), divorced (10.22%), widowed (5.02%), or in informal relationships (7.06%). In terms of work experience, the most common ranges were 31–40 years (33.64%, *n* = 181) and 21–30 years (29.37%, *n* = 158). A small proportion (3.16%) reported less than one year of total work experience. At their current workplace, most participants had worked for less than 5 years (25.46%), followed by 10–20 years (22.68%) and 5–9 years (12.45%). A minority (3.16%) had been employed at the same institution for over 40 years.

Participants were employed in a variety of healthcare settings. The most frequent workplaces were outpatient care facilities (38.29%), medical wards (36.25%), and surgical units (18.77%). A total of 10.59% were employed in intensive care units and emergency departments (ICU/ED), and 12.83% worked in long-term care.

Most respondents were employed on a permanent contract (94.98%). Approximately half (50.74%) worked 168–250 h per month, while 45.72% worked fewer than 168 h. A shift-work system reported by 72.49% of participants. The majority (63.38%) were employed in a single workplace, while 31.60% worked in two facilities simultaneously. Descriptive characteristics of the sample (sex, age, place of residence, education, marital status, job tenure, tenure at the current workplace, and place of work) are presented in Table 1.

**Table 1:** Sociodemographic and professional characteristics of the respondents (*n* = 538).

Variable	Category	*n*	%
Sex	Female	520	96.65
	Male	18	3.35
Age	22–30 years	50	9.29
	31–40 years	67	12.45
	41–50 years	171	31.78
	51–60 years	216	40.15
	>60 years	34	6.32
Place of residence	Rural area	125	23.23
	Urban area	413	76.77
Education	Secondary medical education	144	26.77
	Bachelor of science in nursing	212	39.41
	Master of science in nursing	182	33.83
Marital status	Single	55	10.22
	Married	363	67.47
	Divorced	55	10.22
	Widowed	27	5.02
	Informal relationship	38	7.06
Job tenure	<1 year	17	3.16
	1–10 years	81	15.06
	11–20 years	67	12.45
	21–30 years	158	29.37
	31–40 years	181	33.64
	>40 years	34	6.32
Seniority in current workplace	<5 years	137	25.46
	5–9 years	67	12.45
	10–20 years	122	22.68
	21–30 years	98	18.22
	31–40 years	97	18.03
	>40 years	17	3.16
Place of work	Conservative wards	195	36.25
	Surgical wards	101	18.77
	ICU/emergency department	57	10.59
	Long-term care	69	12.83
	Outpatient care	206	38.29

Percentages may not total 100% due to rounding or multiple responses.

## 3 Measures and instruments

The study used a self-developed questionnaire and three standardized tools: the Hospital Anxiety and Depression Scale—Modified (HADS-M) by Zigmond and Snaith (55), the Job Satisfaction Scale (SSP) by Zalewska (53), and the Satisfaction with Life Scale (SWLS) by Diener et al. (54).

The original questionnaire included 34 items. Section I (items 1–12) addressed sociodemographic and occupational variables. Section II (items 13–22) explored emotional responses related to professional duties. Section III (items 23–34) focused on perceived occupational stress and its effects on personal and professional life.

The HADS-M consists of 16 items that assess three emotional domains: anxiety, depression, and irritability/anger. It is a modified version of the original HADS by Zigmond and Snaith (55). The Polish adaptation was developed by Majkowicz (56). The irritability/anger subscale was added based on an unpublished version used in clinical research. Although HADS-M was originally designed for somatic patients, it has also been applied in population studies, including research among nurses, despite lacking formal validation in this professional group (57) and aggressive behaviors were not assessed. Each subscale uses a 4-point Likert scale (0–3). Scores range from 0–21 for anxiety and depression, and 0–6 for anger. Interpretation thresholds: 0–7 = normal, 8–10 = borderline, ≥11 = possible disorder (58). In this study, Cronbach’s alpha values were: anxiety −0.84, depression −0.863, anger −0.81, confirming good internal consistency.

Job Satisfaction Scale (SSP). This instrument evaluates the cognitive aspect of general job satisfaction and includes five items rated on a 7-point Likert scale (1 = strongly disagree to 7 = strongly agree). A higher score indicates greater job satisfaction. In this study, Cronbach’s alpha was 0.89, consistent with the original validation (53).

Satisfaction with Life Scale (SWLS). This tool assesses global life satisfaction using five items scored on a 7-point Likert scale. Total scores range from 5 to 35, with higher scores indicating greater life satisfaction. The Polish adaptation was developed by Juczyński (59). Reliability coefficients were: original version −0.87 (54), Polish adaptation −0.72 (59), current study −0.89.

### 3.1 Statistical analysis

Data collected from 538 nurses employed in various healthcare facilities were subjected to descriptive and inferential analyses using Statistica v.13.0 (TIBCO Software Inc., 2017) and Microsoft Excel. Categorical variables are presented as *n* (%) and scale scores as mean (SD) or median (IQR), as appropriate. Because summed scale scores can deviate from normality, associations were examined with Spearman’s rank-order correlation (*ρ*). To identify independent predictors of depressive symptoms, anxiety, and irritability/anger symptoms, separate multiple linear regression models were fitted for each dependent variable with SWLS and SSP total scores as predictors. Model assumptions (linearity, normality and homoscedasticity of residuals) were checked via residual diagnostics; multicollinearity was assessed with VIF; influential observations were screened using Cook’s distance. Where heteroscedasticity was detected, heteroscedasticity-robust standard errors (HC3) were used. Standardized coefficients (*β*) with 95% confidence intervals were reported. Internal consistency was evaluated with Cronbach’s alpha. All tests were two-tailed with *α* = 0.05. *Post hoc* power analysis (G*Power 3.1) for a two-tailed correlation (α = 0.05; *n* = 538) indicated power > 0.999 to detect a medium effect (*ρ* = 0.30); the minimum detectable correlation at 80% power was |ρ| ≈ 0.12 (≈0.14 for 90% and ≈0.155 for 95%).

### 3.2 Ethical considerations

The study was conducted in accordance with the Declaration of Helsinki (1975, revised in 2013) (60). All participants gave informed consent, and anonymity was maintained throughout the research. Participants were informed of the study’s purpose and their right to withdraw at any time without consequences. According to Polish regulations and institutional guidelines, anonymous non-interventional survey-based studies that do not involve sensitive personal data or direct health interventions do not require ethics committee approval. These studies are not classified as medical experiments under Article 21 of the Polish Act of December 5, 1996, on the professions of physician and dentist (Dz.U.2019.537 j.t.). Contact details were provided for participants in case of additional questions or concerns.

## 4 Results

A total of 538 nurses employed in various healthcare settings participated in the study. The results are presented in the following order: general psychological symptoms and work-related stress, scale assessments, correlation analyses, and regression modeling. All statistical analyses were performed using Statistica v.13.0 (77) and Microsoft Excel.

### 4.1 Psychological symptoms and emotional states related to work

A large proportion of respondents reported feeling emotionally exhausted due to work (86.25%) and physically fatigued (52.97%). Additionally, 36.80% of participants declared feeling tired upon waking, triggered by the mere thought of going to work.

Work-related anxiety was most frequently reported several times per month (39.96%), several times per week (28.07%), or daily (12.45%). A total of 19.52% of respondents reported no such emotions. Regarding irritability/anger symptoms, 44.61% experienced them several times per month, 31.23% several times per week, and 11.34% daily. No such experiences were reported by 12.83% of participants.

Work satisfaction was experienced several times a month by 41.45% of nurses, several times a week by 27.32%, and daily by 16.73%. A lack of job satisfaction was reported by 14.50% of respondents.

Work errors caused by excessive workload occurred most frequently several times per year (55.58%), less often several times a month (9.85%), once a week (1.86%), or daily (0.74%). A total of 31.97% declared never making such errors. 15.43% reported having taken sick leave due to psychological overload, while 84.57% had not used this form of absence.

### 4.2 Work-related stress and its sources

Workplace stress was common among respondents. Daily stress was reported by 32.90% of participants, several times per week by 32.16%, and several times per month by 32.71%.

The most frequently reported sources of occupational stress included time pressure and work urgency (72.86%) and staff shortages (71.00%). Other reported causes included responsibility for patient health and life (51.86%), lack of supervisor support (44.98%), low pay (42.75%), and poor work organization (37.73%). Additional stressors were contact with patient death (29.74%), lack of breaks (28.25%), fear of infection (28.25%), shift work (26.39%), exposure to harmful factors (25.65%), and limited opportunities for professional development (10.04%). Detailed data are presented in Table 2.

**Table 2:** Distribution of responses to the job satisfaction scale (SSP) (*n* = 538).

Item	1. Strongly disagree		2. Disagree		3. Slightly disagree		4. Neutral		5. Slightly agree		6. Agree		7. Strongly agree	
	*n*	%	*n*	%	*n*	%	*n*	%	*n*	%	*n*	%	*n*	%
1. In many ways, my work is close to ideal	76	14.13	125	23.23	112	20.82	123	22.86	71	13.20	25	4.65	6	1.12
2. I have great conditions at work	76	14.13	128	23.79	109	20.26	109	20.26	91	16.91	20	3.72	5	0.93
3. I am satisfied with the work	43	7.99	76	14.13	98	18.22	128	23.79	128	23.79	59	10.97	6	1.12
4. So far, I have achieved what I aimed for at work	33	6.13	72	13.38	81	15.06	125	23.23	162	30.11	57	10.59	8	1.49
5. If I had to decide again, I would choose the same job	74	13.75	55	10.22	53	9.85	130	24.16	99	18.40	87	16.17	40	7.43

Responses were recorded on a 7-point Likert scale ranging from “1 – strongly disagree” to “7 – strongly agree.” Percentages may not sum to 100% due to rounding.

### 4.3 HADS-M, SSP, and SWLS scale results

According to the HADS-M scale results, 46.65% of respondents occasionally experienced tension and irritability, while 49.07% reported symptoms of depression. Internal anxiety was declared by 44.80% of participants. Sad thoughts occurred in 39.22% of respondents, while 46.10% reported feeling cheerful and positive at times. Conversely, 42.57% stated they sometimes felt irritated and angry; anger symptoms were reported rarely (34.76%) or sometimes (32.53%). Detailed HADS-M results are presented in Tables 3, 4.

**Table 3:** Distribution of responses to the HADS-M irritability/anger symptoms subscale (*n* = 538).

Item	Response	*n*	%
1. I felt tense or irritated	Most of the time	74	13.75
	A lot of the time	187	34.76
	Occasionally	251	46.65
	Not at all	26	4.83
2. I still enjoy things that used to make me happy	Definitely the same	163	30.30
	Not quite the same	250	46.47
	Only a little	107	19.89
	Not at all	18	3.35
3. I felt a frightening feeling, as if something terrible was about to happen	Very clearly	73	13.57
	Clearly	158	29.37
	Slightly	146	27.14
	Not at all	161	29.93
4. I can laugh and see the funny side of events	Same as before	162	30.11
	Now not so much as before	220	40.89
	Much less than before	151	28.07
	Not at all	5	0.93
5. I’m plagued by sad thoughts	Most of the time	38	7.06
	A lot of the time	151	28.07
	Occasionally but not very often	211	39.22
	Rarely	138	25.65
6. I feel happy and cheerful	Not at all	17	3.16
	Not often	129	23.98
	Sometimes	248	46.10
	Most of the time	144	26.77
7. I can sit still and feel relaxed	Definitely yes	0	0.00
	Usually	0	0.00
	Often	538	100.00
	Not at all	0	0.00
8. I feel like I am in a ‘mental hole’	All the time	29	5.39
	Very often	142	26.39
	Occasionally	264	49.07
	Not at all	103	19.14
9. I have an alarming feeling, as if something is shaking inside me	Not at all	185	34.39
	From time to time	241	44.80
	Quite often	94	17.47
	Very often	18	3.35
10. I stopped taking interest in my external appearance	Completely stopped	10	1.86
	Do not take care as I should	136	25.28
	Not able as before	107	19.89
	As always	285	52.97
11. I cannot sit still internally	To a very large extent	41	7.62
	To a large extent	143	26.58
	Rarely	205	38.10
	I can sit	149	27.70
12. I look forward to various things	As much as before	135	25.09
	Less than before	253	47.03
	Definitely less than usual	139	25.84
	Not at all	11	2.04
13. I have a sudden feeling of panic anxiety	Very often	23	4.28
	Quite often	104	19.33
	Not very often	192	35.69
	Not at all	219	40.71
14. I can enjoy a good book, an TV program	Very often	123	22.86
	Quite often	182	33.83
	Not very often	207	38.48
	Not at all	26	4.83
15. There have been times in the past week when I have exploded with anger	Often	70	13.01
	Sometimes	175	32.53
	Rarely	187	34.76
	Not at all	106	19.70
16. There were times when I got upset and angry	Often	134	24.91
	Sometimes	229	42.57
	Rarely	154	28.62
	Not at all	21	3.90

Responses are based on the modified hospital anxiety and depression scale (HADS-M), including anxiety, depression, and irritability/anger subscales. Percentages may not total 100% due to rounding.

**Table 4:** Distribution of responses to the satisfaction with life scale (SWLS) (*n* = 538).

Item	1. Strongly disagree		2. Disagree		3. Slightly disagree		4. Neutral		5. Slightly agree		6. Agree		7. Strongly agree	
	*n*	%	*n*	%	*n*	%	*n*	%	*n*	%	*n*	%	*n*	%
1. In many ways, my life is close to perfect.	43	7.99	106	19.70	119	22.12	171	31.78	94	17.47	5	0.93	0	0.00
2. The conditions of my life are excellent.	24	4.46	95	17.66	140	26.02	164	30.48	108	20.07	7	1.30	0	0.00
3. I am satisfied with my life.	10	1.86	44	8.18	114	21.19	137	25.46	189	35.13	40	7.43	4	0.74
4. In my life I have achieved the most important things I wanted	15	2.79	63	11.71	92	17.10	126	23.42	187	34.76	49	9.11	6	1.12
5. If I could live my life again, I would hardly want to change anything.	64	11.90	144	26.77	101	18.77	88	16.36	116	21.56	21	3.90	4	0.74

Data are based on the satisfaction with life scale (SWLS), which uses a 7-point Likert scale ranging from “1 – strongly disagree” to “7 – strongly agree.” Percentages may not total 100% due to rounding.

The Job Satisfaction Scale (SSP) results revealed notable variation. For example, the statement “In many ways my job is close to ideal” received disagreement from 23.23% of participants, a neutral response from 22.86%, and partial disagreement from 20.82%. The full distribution of responses is provided in Table 5.

**Table 5:** Relationship between levels of depression, anxiety and irritability/anger symptoms and job satisfaction (*n* = 538).

Symptom category		Job satisfaction scale (SSP)						Spearman’s rank correlation
		Mean ± SD	Median [Q25–Q75]	Min.–Max.	Confidence interval		Stand error.	
					−95.00%	+95.00%		
Level of depressive symptoms	No symptoms (*n* = 216)	M = 21.55, SD = 5.6	Mdn = 21.5, IQR = 18–26	5–35	20.80	22.30	0.38	ρ = −0.48; *p* < 0.001
	Subclinical (*n* = 135)	M = 17.6, SD = 5.7	Mdn = 18, IQR = 14–21	5–31	16.63	18.57	0.49	
	Clinical (*n* = 187)	M = 14.44, SD = 5.59	Mdn = 14, IQR = 10–19	5–28	13.64	15.25	0.41	
Level of anxiety symptoms	No symptoms (*n* = 273)	M = 21.05, SD = 5.83	Mdn = 21, IQR = 18–25	5–35	20.35	21.74	0.35	ρ = −0.5; *p* < 0.001
	Subclinical (*n* = 131)	M = 16.68, SD = 5.16	Mdn = 17, IQR = 13–21	5–26	15.79	17.57	0.45	
	Clinical (*n* = 134)	M = 13.43, SD = 5.31	Mdn = 14, IQR = 10–17	5–28	12.53	14.34	0.46	
Level of irritability/anger symptoms	No symptoms (*n* = 279)	M = 20.59, SD = 6.01	Mdn = 21, IQR = 17–25	5–35	19.88	21.30	0.36	ρ = −0.4; *p* < 0.001
	Subclinical (*n* = 194)	M = 15.77, SD = 5.53	Mdn = 16, IQR = 12–20	5–28	14.99	16.56	0.40	
	Clinical (*n* = 65)	M = 14.26, SD = 6.04	Mdn = 14, IQR = 10–19	5–27	12.76	15.76	0.75	

Spearman’s correlation analysis. Differences in job satisfaction across groups are presented as mean ± SD, median [Q25–Q75], range, and 95% confidence intervals. All *p*-values < 0.001.

For the Satisfaction with Life Scale (SWLS), most respondents selected neutral answers to the statements “My life is close to ideal” (31.78%) and “The conditions of my life are excellent” (30.48%). Detailed results are presented in Table 6.

**Table 6:** Relationship between frequency of experiencing work-related stress and levels of depression, anxiety and irritability/anger symptoms (*n* = 538).

Symptom category		Frequency of experiencing stressful situations at work						Spearman’s rank correlation
		Never or a few times per month (*n* = 188)		Several times per week (*n* = 173)		Daily (*n* = 177)		
		*n*	%	*n*	%	*n*	%	
Level of depressive symptoms	No symptoms	118	62.77	58	33.53	40	22.60	ρ = 0.4; *p* < 0.001
	Subclinical	48	25.53	49	28.32	38	21.47	
	Clinical	22	11.70	66	38.15	99	55.93	
Level of anxiety symptoms	No symptoms	132	70.21	74	42.77	67	37.85	ρ = 0.28; *p* < 0.001
	Subclinical	35	18.62	46	26.59	50	28.25	
	Clinical	21	11.17	53	30.64	60	33.90	
Level of irritability/anger symptoms	No symptoms	134	71.28	75	43.35	70	39.55	ρ = 0.28; *p* < 0.001
	Subclinical	47	25.00	75	43.35	72	40.68	
	Clinical	7	3.72	23	13.29	35	19.77	

Based on frequency distribution of work-related stress levels and symptom categories. Spearman’s correlation used; all *p*-values < 0.001.

### 4.4 Correlations between variables

Spearman’s rank-order correlation analyses showed statistically significant negative associations between life satisfaction and symptoms of depression (*ρ* = −0.56; *p* < 0.001), anxiety (ρ = −0.55; *p* < 0.001), and (ρ = −0.38; *p* < 0.001). Similar correlations were found between job satisfaction and these symptoms: depression (ρ = −0.48), anxiety (ρ = −0.50), and (ρ = −0.40), all significant at *p* < 0.001. Results are summarized in Table 7.

**Table 7:** Relationship between frequency of experiencing work-related stress and levels of life and job satisfaction (*n* = 538).

Scale	Frequency of experiencing stressful situations at work	Descriptive statistics						Spearman’s rank correlation
		Mean ± SD	Median [Q25–Q75]	Min.–Max.	Confidence interval		Stand error.	
					−95.00%	+95.00%		
Satisfaction with life scale (SWLS)	Never or a few times per month (*n* = 188)	M = 19.97, SD = 4.69	Mdn = 20, IQR = 17–23	6–29	19.30	20.65	0.34	ρ = −0.26; *p* < 0.001
	Several times per week (*n* = 173)	M = 17.62, SD = 4.85	Mdn = 18, IQR = 14–21	7–29	16.90	18.35	0.37	
	Daily (*n* = 177)	M = 16.86, SD = 5.16	Mdn = 17, IQR = 13–21	5–28	16.09	17.62	0.39	
Job satisfaction scale (SSP)	Never or a few times per month (*n* = 188)	M = 20.86, SD = 6.02	21 [17–25.5]	5–34	20.00	21.73	0.44	ρ = −0.32; *p* < 0.001
	Several times per week (*n* = 173)	M = 17.13, SD = 6.14	Mdn = 17, IQR = 14–21	5–35	16.21	18.05	0.47	
	Daily (*n* = 177)	M = 16.07, SD = 6.05	Mdn = 16, IQR = 12–20	5–33	15.18	16.97	0.45	

The table presents descriptive statistics and Spearman’s rank correlation coefficients for the relationship between the frequency of experiencing stressful situations at work and levels of life satisfaction (SWLS) and job satisfaction (SSP). A significant negative correlation was found in both cases (*p* < 0.001), indicating that more frequent work-related stress is associated with lower satisfaction levels.

### 4.5 Predictors of psychological functioning—regression analysis

Multiple linear regression models were constructed to identify predictors of depression, anxiety, and symptoms. Total scores from the SWLS and SSP were included as independent variables in each model. All three models reached statistical significance (*p* < 0.001). The highest explanatory power was observed in the depression model (R^2^ = 0.452), followed by anxiety (R^2^ = 0.324) and (R^2^ = 0.237). Regression results are summarized in Table 8.

**Table 8:** Multiple regression models predicting depression, anxiety, and irritability/anger symptoms (*n* = 538).

Symptom level	*R*	*R^2^*	Adjusted *R^2^*	F(df)	*p*-value	*β*(SWLS)	*p*(SWLS)	*β*(SSP)	*p*(SSP)
Level of depressive symptoms	0.672	0.452	0.45	*F*(2, 535)	< 0.001	−0.429	< 0.001	−0.315	< 0.001
Level of anxiety symptoms	0.569	0.324	0.321	*F*(2, 535)	< 0.001	−0.332	< 0.001	−0.299	< 0.001
Level of irritability/anger symptoms	0.487	0.237	0.234	*F*(2, 535)	< 0.001	−0.231	< 0.001	−0.307	< 0.001

All regression models were statistically significant (*p* < 0.001). SWLS – Satisfaction With Life Scale; SSP – Job Satisfaction Scale.

### 4.6 Reliability of measurement instruments

Internal consistency of the applied scales was assessed using Cronbach’s alpha. The HADS subscales yielded the following values: HADS-Anxiety (*α* = 0.868; after removing item 7), HADS-Depression (α = 0.863), and HADS-Anger subscale (α = 0.813). The Satisfaction with Life Scale (SWLS) reached α = 0.86, and the Job Satisfaction Scale (SSP) α = 0.89. These values indicate high psychometric reliability. Reliability coefficients are presented in Table 9.

**Table 9:** Internal consistency (Cronbach’s alpha) of the applied scales.

Scale	Cronbach’s alpha (α)
HADS-anxiety (after removing item 7)	0.868
HADS-depression	0.863
HADS-irritability/anger	0.813
Satisfaction with life scale (SWLS)	0.860
Job satisfaction scale (SSP)	0.890

Cronbach’s alpha values above 0.80 indicate good internal consistency of the applied instruments, supporting their psychometric reliability.

### 4.7 Ethical considerations

All participants provided informed consent. According to national regulations and institutional policies, ethical approval was not required for this type of non-interventional, anonymous online survey.

## 5 Discussion

The findings of this study allow for a multidimensional analysis of the severity of depression, anxiety, and irritability/anger symptoms among nurses, as well as their relationship with job and life satisfaction. The nursing profession is associated with substantial physical and mental burdens. Occupational stress—resulting from interactions with patients and their families, staffing shortages, shift work, and interpersonal dynamics—is a constant element of nurses’ professional reality. These factors promote the development of intensified anxiety and depressive symptoms, driven by prolonged emotional strain, a sense of helplessness, and exposure to death and suffering.

A high prevalence of anxiety among nurses has been confirmed in previous studies. For instance, Sharif et al. (61) reported that 89.7% of respondents experienced anxiety, with 73% describing it as moderate or severe. These data are consistent with the current findings, where 12.45% of nurses experienced anxiety daily, 28.07% several times per week, and 39.96% several times per month. Similarly, Huang et al. (11) indicated that longer work experience may increase the risk of anxiety disorders, while Maharaj et al. (62) reported symptoms in 41.2% of nurses.

Depression, one of the most common mental disorders, also affects nursing personnel. Constant exposure to illness, death, and clinical responsibility may lead to chronic mood deterioration. In this study, 6% of participants reported experiencing depressive symptoms daily, 26.39% very frequently, and nearly half (49.07%) declared sporadic symptoms. Anxiety and depression are currently among the most frequently diagnosed mental health conditions (63). According to the World Health Organization, nurses are particularly vulnerable to the negative effects of occupational stress (24). This is supported by Angermeyer et al. (64), who found a high prevalence of anxiety, depression, and fatigue among nurses. Wang et al. (65) also emphasized that workplace-related factors significantly influence the development of psychopathological symptoms.

International studies have reported that the prevalence of anxiety and depressive symptoms among nurses ranges from 11 to 80%, depending on the country and methodology (62). In our study, 34.76% met the HADS-M clinical cut-off for depressive symptoms and 24.91% met the clinical cut-off for anxiety symptoms, which is consistent with the mid-to-upper range of prior estimates.

Shift work, performed by 72.49% of participants, has been identified as a factor that disrupts circadian rhythms and burdens mental health. Several studies [e.g., Maharaj et al. (62), Cheung et al. (66), Sharif et al. (61), and Motta de Vasconcelos et al. (67)] have demonstrated a relationship between shift work and depression. Additionally, a lack of social support and emotional connections can increase the risk of mood disorders (65, 67).

In light of the above findings, implementing preventive programs and psychological interventions in the workplace appears justified. Literature suggests that mindfulness-based interventions may help enhance psychological resilience and reduce stress (68).

Irritability/anger symptoms, frustration, and emotional aggression—both internalized and externally expressed—also warrant attention. Only 12.83% of respondents in this study reported an absence of such emotions. The most commonly reported frequency of anger was several times per month (44.61%).

These findings are consistent with those of Wyderka and Niedzielska (69), who found that 40% of nurses had difficulties with emotional regulation. Cheung et al. (66) noted that nurses are more likely to experience emotional aggression than other professional groups. Mosiołek et al. (70) reported that 66.7% of respondents had experienced verbal aggression and 50% had experienced physical aggression from patients.

The impact of emotional overload on nurses’ mental health is therefore significant. In terms of occupational satisfaction, only 5.57% of participants described their job as fully satisfying, while more than half (58.18%) reported varying degrees of dissatisfaction.

Nonetheless, 44.61% of respondents indicated they would choose the nursing profession again. In a study by Ostrowicka et al. (71), only 5% of participants reported a complete lack of job satisfaction. Factors influencing satisfaction included workplace relationships, employment stability, and working conditions.

Kunecka (72) and Gawęda et al. (73) have emphasized that a positive team atmosphere and a sense of meaning in work enhance job satisfaction. Lubrańska (74) found a strong correlation between job satisfaction and life satisfaction. Siemiginowska et al. (75) and Mroczkowska et al. (76) highlighted that shift work intensifies work–life conflict, although it does not always directly reduce overall satisfaction.

In summary, our findings confirm that nurses’ psychological well-being is strongly influenced by working conditions, interpersonal relationships, and workplace organization. Therefore, healthcare facility administrators should implement comprehensive strategies to promote the mental health of nursing personnel. Particular emphasis should be placed on improving the work environment, reducing occupational stressors, and preventing workplace phenomena such as bullying. Proper interventions may help reduce symptoms of anxiety and depression, and anger symptoms, thereby enhancing occupational well-being.

### 5.1 Limitations of the study

Despite the statistically significant findings, this study has several limitations that must be considered when interpreting and generalizing the results.

First, the use of purposive sampling and an online survey format may have influenced the sample structure. The study primarily included individuals active on social media, potentially excluding participants less familiar with digital technologies. It is also possible that the sample was overrepresented by individuals more motivated to share emotional experiences, as evidenced by a higher proportion of older respondents with longer work experience.

Second, the cross-sectional design of the study does not allow for causal inferences. The results reflect co-occurring variables at a single point in time, without the possibility of assessing their temporal dynamics. Moreover, although standardized and validated self-report tools were used, there remains a risk of subjectivity, anchoring bias, or socially desirable responses.

It is also important to note that the study focused exclusively on nurses working in Poland. Thus, caution should be exercised when attempting to generalize these findings to other professional or international populations that may operate under different systemic conditions. We did not collect province/voivodeship, formal specialty status, or job grade (e.g., ward/charge vs. staff), which limits geographic and professional subgroup analyses.

Nevertheless, this study provides a valuable contribution to understanding the mental health, well-being, and working conditions of nurses, and underscores the need for further research in this field.

## 6 Conclusion

Nurses experience considerable psychological burden, which contributes to the intensification of depressive, anxiety, and irritability/anger symptoms, as well as a reduction in life and job satisfaction. The most significant factors negatively affecting their well-being include shift work, excessive workload, lack of organizational support, and frequent exposure to stressful situations.

The results revealed statistically significant associations: the more intense the emotional symptoms, the lower the levels of life and job satisfaction. This highlights the critical role of working conditions and interpersonal relationships in maintaining the mental health of this professional group.

The findings emphasize the need to implement strategies that support the mental health of nursing personnel. These include emotional aggression prevention, the development of psychoeducational programs, and organizational changes aimed at reducing occupational stress. Ensuring the psychological well-being of nursing teams may not only improve the quality of patient care but also enhance the overall functioning of the healthcare system.

## Data Availability

The original contributions presented in the study are included in the article/supplementary material, further inquiries can be directed to the corresponding author.
